# Polar Interactions at the Dimer–Dimer Interface of Methionine Adenosyltransferase MAT I Control Tetramerization

**DOI:** 10.3390/ijms222413206

**Published:** 2021-12-08

**Authors:** Gabino Francisco Sánchez-Pérez, María Ángeles Pajares

**Affiliations:** 1 Instituto de Investigaciones Biomédicas Alberto Sols (CSIC-UAM), Arturo Duperier 4, 28029 Madrid, Spain; gabino.sanchez.perez@gmail.com; 2 Hudson River Biotechnology, Nieuwe Kanaal 7V, 6709 PA Wageningen, The Netherlands; 3 Centro de Investigaciones Biológicas Margarita Salas (CSIC), Ramiro de Maeztu 9, 28040 Madrid, Spain

**Keywords:** association mechanism, cooperativity, dimer/tetramer ratio, methionine cycle, oligomerization, polar interactions, S-adenosylmethionine synthesis, tripolyphosphatase activity

## Abstract

Catalytic MATα1 subunits associate into kinetically distinct homo-dimers (MAT III) and homo-tetramers (MAT I) that synthesize S-adenosylmethionine in the adult liver. Pathological reductions in S-adenosylmethionine levels correlate with MAT III accumulation; thus, it is important to know the determinants of dimer–dimer associations. Here, polar interactions (<3.5 Å) at the rat MAT I dimer–dimer interface were disrupted by site-directed mutagenesis. Heterologous expression rendered decreased soluble mutant MATα1 levels that appeared mostly as dimers. Substitutions at the B1–B2 or B3–C1 β-strand loops, or changes in charge on helix α2 located behind, induced either MAT III or MAT I accumulation. Notably, double mutants combining neutral changes on helix α2 with substitutions at either β-strand loop further increased MAT III content. Mutations had negligible impact on secondary or tertiary protein structure, but induced changes of 5–10 °C in thermal stability. All mutants preserved tripolyphosphatase activity, although AdoMet synthesis was only detected in single mutants. Kinetic parameters were altered in all purified proteins, their AdoMet synthesis V_max_ and methionine affinities correlating with the association state induced by the corresponding mutations. In conclusion, polar interactions control MATα1 tetramerization and kinetics, diverse effects being induced by changes on opposite β-sheet loops putatively leading to subtle variations in central domain β-sheet orientation.

## 1. Introduction

S-adenosylmethionine (AdoMet) is a promiscuous compound able to participate in a large variety of reactions that are estimated to be as numerous as those involving ATP. Mostly known for its role as the main cellular methyl donor, its importance goes far beyond, due to the capacity of AdoMet to donate many other groups [1]. Nevertheless, only the highly conserved methionine adenosyltransferases (MATs) catalyze AdoMet synthesis in a two-step reaction that uses methionine and ATP as substrates and requires Mg^2+^ and K^+^ ions [2,3]. In the first part of the reaction, methionine is added to the adenosine moiety of ATP, rendering AdoMet and triphosphate (PPP_i_). Liberation of the methyl donor from the active site then requires PPP_i_ hydrolysis into pyrophosphate and inorganic phosphate. These two activities can be independently measured, a fact that has enabled the characterization of some mutations in the MAT family, leading to proteins with decreased or erased AdoMet synthesis activity, while tripolyphosphatase (PPPase) activity is preserved [4,5,6]. Some of these mutants were identified in the 1970s during newborn screenings for persistent hypermethioninemia (>43 µM methionine in plasma), also named Mudd’s disease [4].

In mammals, there are three MAT genes, named *MAT1A*, *MAT2A* and *MAT2B*. *MAT1A* expression is high in adult hepatocytes, but very low in other cell types and tumor cells (reviewed in [2,3,7]). In contrast, *MAT2A* expression is high in fetal hepatocytes and in most adult and cancer cells [8,9], with *MAT2B* closely following this same expression pattern (reviewed in [2,3,7]). *MAT1A* and *MAT2A* codify for the highly conserved catalytic subunits MATα1 and MATα2 [10], respectively, whereas the *MAT2B* gene encodes the unrelated regulatory MATβ monomer [11]. These proteins display mainly cytoplasmic localization, but they have been also found in the cell nucleus and MATα1 even in the mitochondria [12,13,14]. The structure of MATα subunits is organized in three domains that contribute to a large flat hydrophobic surface, through which they associate into homo-dimers [15,16,17]; those constituted by MATα1 subunits are named MAT III isoenzymes. Two active sites per dimer, opposite one another, are formed by this association with residues of each monomer contributing to catalysis; hence, homo-dimers are the minimum active form of the enzyme. Further associations of MAT III into homo-tetramers, named MAT I isoenzymes, occur by the interaction of two dimers through the central domain of each subunit [15]. In contrast, MATα2 dimers bind a single MATβ subunit to form the hetero-trimeric isoenzyme MAT II [18,19].

Healthy adult hepatocytes contain high levels of MAT I and MAT III; in liver pathology, their content decreases, whereas that of MAT II is enhanced [8,20,21,22]. Analysis of the oligomerization state of MATα1 in biopsies of human liver cirrhosis and in animal models of acute liver intoxication demonstrated that the global decreases in MATα1 protein in these pathologies correlate with cytoplasmic accumulation of the protein as MAT III dimers, therefore changing the MAT I/III ratio in this compartment [21,22]. However, this cytoplasmic oligomerization change is not uniformly found in the few liver pathology models analyzed to date and, in fact, a trend toward MAT I accumulation was described in young Long Evans Cinnamon rats [20], a model of early-stage Wilson disease. Additionally, reductions in cytoplasmic MATα1 content in acute liver intoxication and hepatoma cells are correlated with nuclear accumulation [21,23] which, in turn, are associated with increased nuclear MAT I content and epigenetic methylation levels [21]. Oligomerization changes have also been described among the few human *MAT1A* mutations identified in patients with hypermethioninemia, in which this parameter has been studied (e.g., dimeric R264H) [24].

Oligomerization changes are extremely important for maintaining adequate AdoMet levels for cell function, given the differences in kinetic parameters between the isoenzymes. V_max_ values are high for MAT III, intermediate for MAT I, and low for MAT II, whereas the opposite pattern is found in their methionine affinities [2,3]. Indeed, only S_0.5_^Met^ values for MAT I and MAT II are in the micromolar range within cellular methionine concentrations; for hepatocytes, these are 50–80 μM [25]. Hence, the reduction in AdoMet levels have been detected in several hepatic pathologies as a consequence of decreases in *MAT1A* expression and in the MAT I/III ratio (reviewed in [7,26]). Redox stress, and specifically reductions in the GSH/GSSG ratio that are commonly observed in liver disease, further contribute to this scenario as key regulators of MAT I/III activity, oligomeric state and subcellular localization [21,27,28,29]. In fact, increases in GSSG content inhibit AdoMet synthesis by these isoenzymes [20,21,27,28], favor production of an intrasubunit disulfide that blocks dimer/tetramer exchange [29], displace the MATα1 association state towards dimers and even monomers [27,28], and also induce the nuclear accumulation of MAT I and MATα1 [21]. The consequences are imbalances in the ratio between association states in the cytoplasm and the nucleus that seem directed to favor nuclear AdoMet supply.

Altogether, these data highlight the importance of knowing the mechanisms controlling the association state of MATs. To date, this knowledge is restricted to the important role of central domain cysteines in the association state of several members of the MAT family [30,31,32]. Single substitutions by serine of each of the five cysteines in the rat MATα1 central domain lead to alterations in the dimer/tetramer ratio [15], an effect especially exacerbated in the C69S protein which appears mostly as dimers [32]. Moreover, among these cysteines, C61 is specific of MATα1 subunits and forms an intrasubunit disulfide with C35 in vivo [33]. The production of this disulfide bond is favored by low GSH/GSSG ratios and precludes dimer/tetramer exchange [29]. In this line, we have focused our attention on the rat MAT I dimer–dimer interface and the few polar interactions established at <3.5 Å to maintain the homo-tetramer [15]. Kinetic characterization and analysis of the impact on the dimer/tetramer ratio of the disruption of these interactions revealed the key role of R85 and T63 in sustaining the MAT I homo-tetramer.

## 2. Results and Discussion

**Figure 1:**
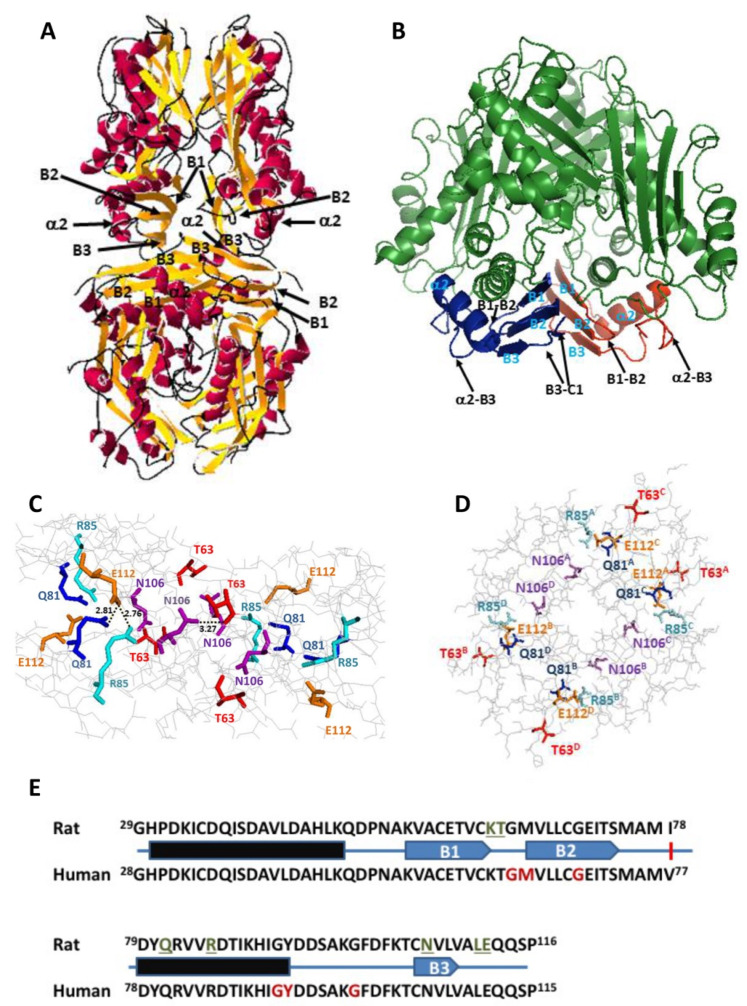
Secondary structure elements and mutated residues involved in polar interactions of <3.5 Å at the dimer–dimer interface of the rat MAT I homo-tetramer. Panel (**A**) shows a cartoon diagram of the MAT I crystal structure (PDB 1QM4) with colored secondary structure elements: α-helices (magenta) and β-strands (orange and connecting loops (black). Elements of the central domain mentioned in the text (helices α1 and α2, β-strands B1, B2 and B3) appear labeled in black. Panel (**B**) depicts a cartoon diagram of the dimer (forest green) with the C61–E112 stretch of subunits colored in red (monomer AA) and blue (monomer B). Secondary structure elements mentioned in the text appear labeled in cyan or indicated by arrows. Panel (**C**) shows a side view of the dimer–dimer interface, with mutated residues highlighted as colored sticks: T63 (red), Q81 (blue), R85 (cyan), N106 (magenta) and E112 (orange). Distances (Å) measured for selected polar interactions are indicated. Panel (**D**) depicts a top view of the C61–E112 stretch with mutated residues colored as in panel (**C**), superscripts indicating the corresponding monomer (A, B, C and D). Panel (**E**) displays a BlastP comparison of the rat and human MATα1 sequences comprising residues 29–116, together with the location of α-helices (black rectangles) and β-strands (blue arrows). Residues involved in the dimer–dimer polar interactions of rat MAT I appear underlined and in green, whereas positions of human mutations identified to date are indicated in red.

The crystal structure of rat MAT I (1QM4) showed the existence of a reduced number of polar interactions within <3.5 Å between upper and lower dimers that stabilize the homo-tetramer [15]. These interactions involve residues on three subunits of the tetramer, each monomer establishing such interactions with both subunits of the opposite dimer; hence, each interaction can be found four times at the dimer–dimer interface. Focusing on one of the upper subunits, the residues implicated are Q81, R85, K62 and T63 of monomer A, whereas those in the lower dimer are E112 and L111 of monomer C and N106 of monomer D (Figure 1). Among them, Q81, E112 and N106 established two such polar interactions each (Table 1). All these residues concentrated into the 29–116 stretch, which is extremely conserved between rat and human MATα1 sequences (Figure 1E), and that together with the 255–289 segment, constitute the central domain of the monomer [15]. Moreover, they are placed on a few secondary structure elements within this central domain, precisely: the B1–B2 loop (K62, T63) connecting β-strands B1 and B2 of the β-sheet; the B3–C1 loop (L111, E112) linking β-strands B3 of the central domain and C1 of the C-terminal domain; the start of β-strand B3 (N106); and helix α2 (Q81, R85) located behind the central domain β-sheet and closer to the protein surface. Importantly, the human MAT I structure (PDB 6SW5) preserves similar distances between equivalent residues at the dimer–dimer interface, although in certain cases, the groups involved in the interactions may vary from those found in rat protein [34].

In order to understand how these few polar interactions contribute to MAT I stability, their systematic disruption was carried out by site-directed mutagenesis. The substitutions were selected to eliminate the groups involved in the interactions, thus: (i) T63V erased the hydroxyl group of threonine; (ii) Q81T introduced a chain lacking amino and carbonyl groups of glutamine; (iii) R85L and R85E suppressed or changed the charge of the arginine lateral chain, respectively; (iv) N106A removed the charged groups of asparagine; and (v) E112L eliminated the negative charge of the glutamic acid. In most cases, the impact caused by the new residue was a slight reduction in the lateral chain size, except for the N106A mutation, which kept the main chain conformation while suppressing most of the side chain [35].

### 2.1. Impact of Mutations on the Production of Soluble Recombinant MATα1

**Figure 2:**
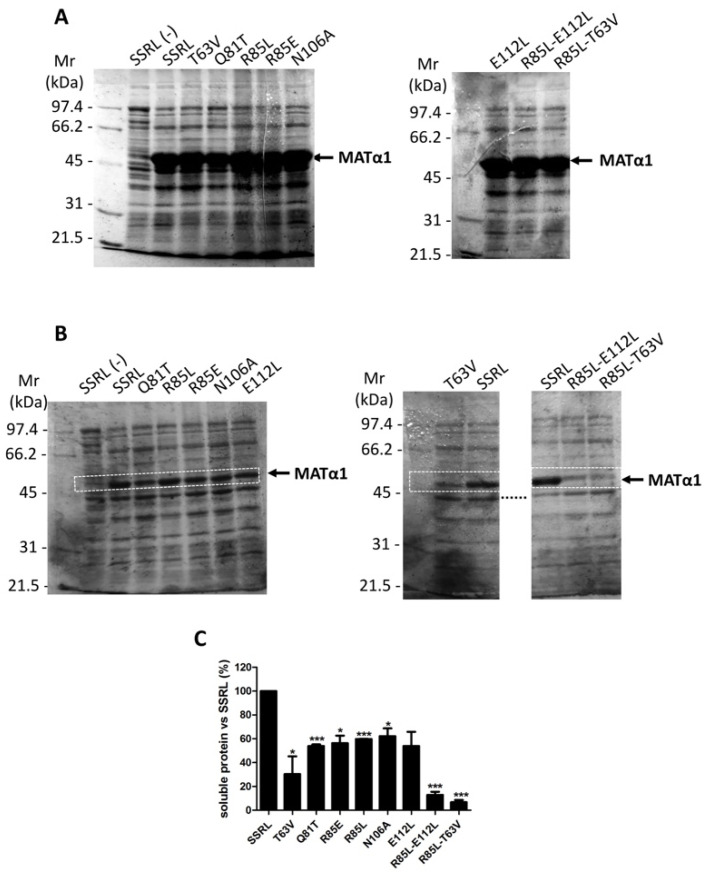
Expression of wild-type and mutant MATα1 in *E. coli*. The plasmids containing the sequence of wild-type MATα1 (SSRL) or the mutants were transformed in *E. coli* and their expression was induced for 3 h with IPTG. Whole lysates (20 μg/lane) and soluble fractions (20 μg/lane) were separated on SDS-PAGE gels and stained with Coomassie Blue. Panel (**A**) shows a typical stained gel of whole lysates from SSRL and each mutant protein, as indicated above. Panel (**B**) displays a typical stained gel of soluble fractions expressing SSRL and mutants. In both panels, a lane including non-induced IPTG fractions (SSRL (-)) is also shown for comparison. The molecular mass of the standards is indicated on the left, and the position of the MATα1 band is on the right of each gel. Dotted lines indicate positions at which lanes have been cropped from the representative gel shown. Panel (**C**) depicts the results (mean ± SEM) of densitometric scanning of MATα1 bands in the soluble fractions for each mutant obtained from two independent experiments. Bands corresponding to MATα1 are indicated with white dotted rectangles in the representative gels shown in panel (**B**). SSRL levels were considered as 100%, and data for the mutants refer to this value. Results were considered significant vs. SSRL when: * *p* ≤ 0.05, *** *p* ≤ 0.001.

Heterologous expression of the wild-type (SSRL) and mutant proteins showed the presence of the corresponding MATα1 forms in whole lysates after IPTG induction (Figure 2A). Densitometric scanning of the stained gels indicated that mean expression levels of SSRL and all mutants reached 23.38 ± 1.53% of the total protein, as previously reported for SSRL [36]. Nevertheless, further fractionation of the lysates into soluble and insoluble fractions (inclusion bodies) showed that all the mutations decreased the amount of soluble protein produced (Figure 2B). Densitometric scanning of the corresponding gels indicated that these reductions were 40–50% for Q81T, R85E, R85L, N106A and E112L, ~70% for T63V, and >90% for the double R85L–E112L and R85L–T63V mutants compared to soluble SSRL protein levels (Figure 2C). Interestingly, a similar analysis carried out with cysteine mutants of the central domain only showed ~90% decrease in soluble C57S protein [32]. Both C57 and T63 residues are located in the β-sheet of the central domain, the former laying at the center of β-strand B1 and the second at the B1–B2 loop. Therefore, it could be postulated that perturbations in this area influence severely protein folding and/or aggregation.

However, double mutants combining substitutions in the B1–B2 or B3–C1 loops with mutations in helix α2 further reduce the solubility of the resulting proteins. Hence, effects on solubility seem to depend on loops lying at both sides of the β-sheet, as well as on helix α2. To date, few human mutations have been identified in this area, including G63V [37], M64K [38], G69S [39,40], G91S [41], Y92H [39,42,43,44], Y92X [45,46], G98S [46] and G98R [4] that occur at residues conserved between human and rat MATα1 and located on β-strand B2, helix α2 and the B1–B2 loop. To the best of our knowledge, only the G98S protein has been studied upon *E. coli* and COS1 expression; however, information about the impact of this mutation on solubility is lacking [46]. Nevertheless, the presence of G63 and M64 in the same conserved block II as rat C57, K62 and T63 [10] suggests that G63V and M64K proteins may also exhibit changes in solubility that need to be confirmed experimentally.

The presence of the mutants in soluble fractions could be also followed by measuring AdoMet synthesis activity, if it is not erased by the substitution performed (Table 2). Comparisons between data with and without IPTG induction were necessary due to the contribution of *E. coli* MAT to AdoMet synthesis in these assays. Values of SSRL activity upon IPTG induction were similar to those previously reported [32,36], whereas those without IPTG induction were lower [36]. Hence, this resulted in a six-fold higher increase in AdoMet synthesis upon SSRL overexpression in the present study, reflecting a lower contribution of the *E. coli* MAT measured without IPTG induction. The expression of most mutants increased AdoMet synthesis activity in the corresponding soluble fractions, except for the double R85L–T63V mutant, which was barely detectable. Comparison with SSRL activity data showed that only the Q81T mutant attained similar AdoMet synthesis levels, whereas reductions above 75% were exhibited by the rest of the mutants. These results suggested the preservation of AdoMet synthesis in all the mutants, although the real impact of the substitutions could not be ascertained in these calculations which used the total protein content. In fact, considering the amount of soluble MATα1 for each mutant (Figure 2C), the Q81T mutation seemed to increase AdoMet synthesis activity vs. SSRL, whereas the rest of the substitutions decreased this parameter. Previous studies also reported that other mutations carried out in this same rat sequence stretch decreased AdoMet synthesis activity to different extents, reaching 30% for C61S, 70% for C69S and C105S, and 95% for C57S [32]. Our results, and those for cysteine mutants, could be expected from the transmission of perturbations in the central domain β-sheet to the loop regulating access to the MAT active site, because both elements are directly linked [15]. Surprisingly, this does not seem to be the case for the human His-tagged G69S mutant expressed in *E. coli* which preserves 100% AdoMet synthesis and PPPase activities [39]. This lack of effect could derive from a more conservative substitution, the influence of the tag, or from the different roles of closely positioned residues during folding (rat ^69^CG^70^ vs. human ^68^CG^69^).

### 2.2. Effects of the Mutations on the Association State

**Figure 3:**
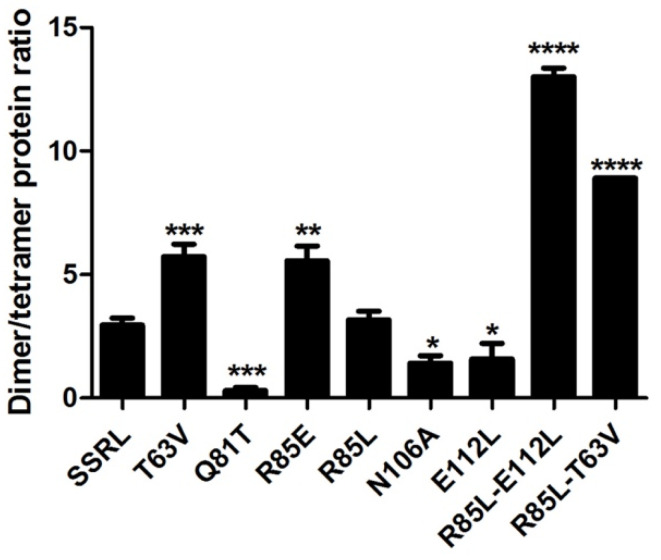
Dimer/tetramer protein ratios of wild-type and mutant MATα1 by hydrophobic chromatography. Soluble fractions of *E. coli* overexpressing wild-type MATα1 (SSRL) and mutants at a fixed protein concentration of 0.5 mg/mL were loaded on phenyl Sepharose columns, and the flowthrough (2 mL) containing the homo-tetramer collected. After extensive washing, elution of the homo-dimer was carried out using 50% (*v*/*v*) DMSO (5 mL). Samples (100 µL) of these fractions were spotted on nitrocellulose for the dot-blot detection of MATα1, and dimer/tetramer ratios (mean ± SEM) were estimated by densitometric scanning of the images, after correction for differences in volume between fractions. Results shown correspond to at least two independent experiments carried out in triplicate. Results were considered significant vs. SSRL when: * *p* ≤ 0.05, ** *p* ≤ 0.005, *** *p* ≤ 0.001, **** *p* < 0.0001.

**Figure 4:**
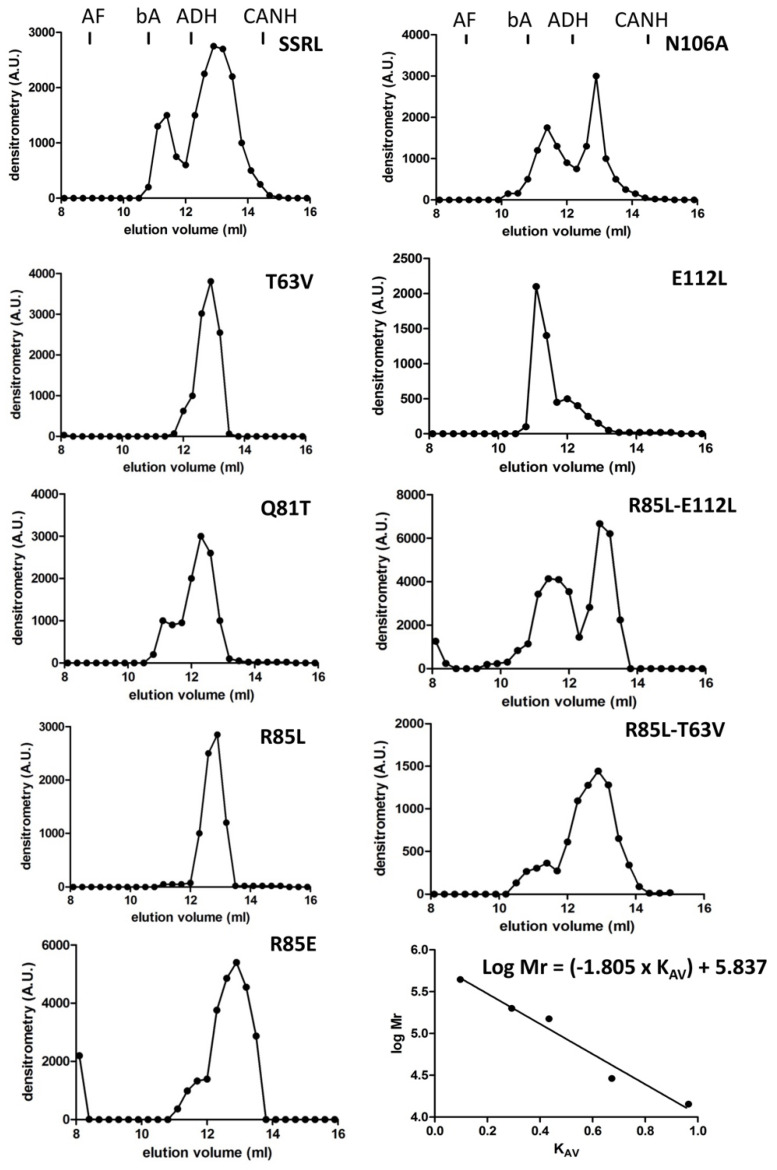
Analytical gel filtration chromatography analysis of soluble wild-type and mutant MATα1. Aliquots (100 µL) of soluble fractions of *E. coli* overexpressing wild-type MATα1 (SSRL) or the mutants at 0.5 mg/mL were loaded on a Superose 12 10/30 HR column for separation of the oligomeric forms. Samples of the collected fractions (300 µL) were used for dot-blot detection using anti-MATα1 antiserum, and the elution profiles were obtained by densitometric scanning of the images. The elution volume of the standards is indicated on the top panels and a representative calibration curve is included (lower right panel). Elution of the protein standards used for column calibration was as follows: blue dextran (7.98 mL), apoferritin (8.92 mL), β-amylase (10.81 mL), alcohol dehydrogenase (12.18 mL), carbonic anhydrase (14.49 mL), lysozyme (17.31 mL) and ATP (17.65 mL).

Impacts of the mutations in the dimer/tetramer ratio were analyzed using the soluble fractions and two chromatographic methods in parallel. On the one hand, analytical gel filtration chromatography (AGFC) allowed separation of the oligomers according to their size, ~210 kDa for homo-tetramers and ~110 kDa for homo-dimers [47], and on the other hand, hydrophobic chromatography on phenyl Sepharose. Tetramers do not bind to these hydrophobic beads, whereas dimers stick strongly to the gel and can be only eluted with 50% (*v*/*v*) DMSO [48]. Previous reports have described that recombinant MAT appears as homo-tetramers and homo-dimers in a protein-concentration-den- pendent equilibrium [49]; therefore, this analysis was carried out using a fixed protein concentration (0.5 mg/mL) which allowed the simultaneous detection of both isoenzyme forms. Compared to SSRL, all the mutations altered the dimer/tetramer ratio, except for R85L, which eliminated the positive charge at this position on helix α2 (Figure 3). Consistent increases in tetramer content were detected for Q81T, N106A and E112L mutations, each of them abolishing two polar interactions. In contrast, R85E, T63V and the double mutants R85L–T63V and R85L–E112L resulted in dimer accumulation. In general, correspondence between AGFC and phenyl Sepharose dimer/tetramer estimates was observed for those mutants with high or intermediate expression levels in the soluble fraction (Figure 4). The importance of polar interactions in MATα1 association state is not restricted to the dimer–dimer interface because, according to the crystal structures, there are also a few such interactions at the monomer–monomer interface [15]. One of them involves residues R265 and E58 of rat MAT III and is conserved in the *E. coli* and human counterparts; this interaction seems to have a role in K^+^ activation. Mutants disrupting this interaction have been identified in human dominant mid-hypermethioninemia and, precisely, the R264H mutant was characterized as a dimer [24]. However, disparities concerning the association state were found when the equivalent rat (R265H) and *E. coli* (R244H) mutants were studied, which were described as monomers and tetramers, respectively [5,50]. Surprisingly, the rat R265H monomer preserved PPPase activity and was able to associate with wild-type MATα1 subunits, the mixed dimer recovering AdoMet synthesis activity [5]. Nevertheless, this residue does not belong to the central domain, although the polar interaction disrupted by the mutations is established with E58, which locates on β-strand B2 of the central domain.

**Figure 5:**
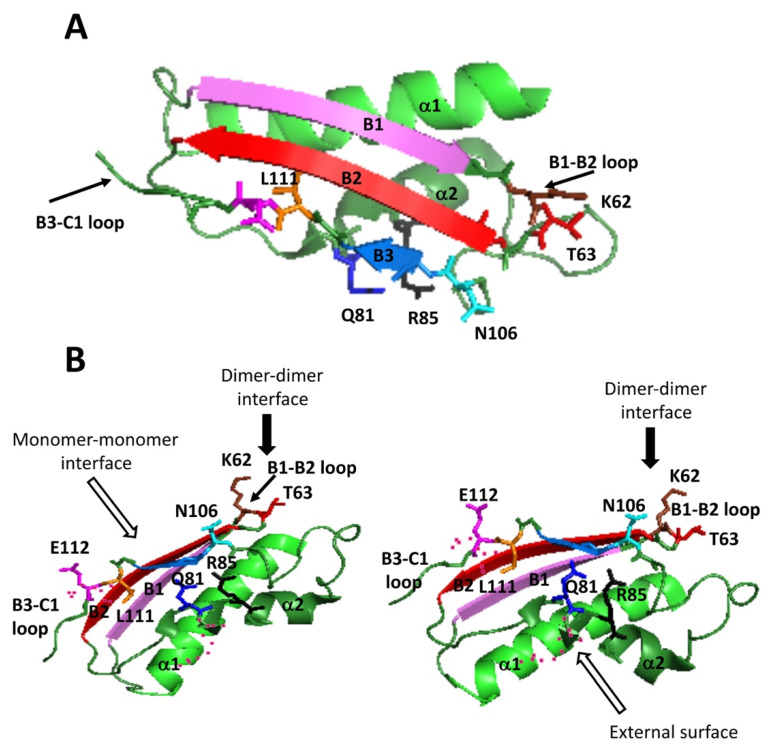
Views of the structural elements of a monomer contributing to the dimer–dimer interface of the rat MAT I homo–tetramer. The figure depicts several views of secondary structure elements within the H30-P116 segment from the MATα1 monomer central domain which contribute to the dimer–dimer interface: helix α1 (green), helix α2 (forest green), β-strand B1 (pink), β-strand B2 (red), β-strand B3 (blue marine), B1–B2 and B3–C1 loops (black arrows). Residues involved in polar interactions of <3.5 Å are represented as sticks: K62 (chocolate), T63 (red), Q81 (blue), R85 (black), N106 (cyan), L111 (orange) and E112 (magenta). Panel (**A**) displays a view from the monomer–monomer interface of this part of the central domain. Panel (**B**) illustrates side views of the same region with structural elements contributing to dimer–dimer interface oriented to the top.

Interestingly, all the residues mutated in our study belong to the same stretch containing the five cysteine residues present in the central domain (C35, C57, C61, C69 and C105), including C35 and C61, which form the intrasubunit disulfide precluding dimer/tetramer interconversion [29,33]. In fact, results from previous mutagenesis studies carried out in *E. coli* MAT and rat MATα1 already showed the importance of cysteines in the central domain β-sheet for dimer–dimer association. In *E. coli* MAT, C89S and C89A substitutions induced dimer/tetramer equilibrium in an otherwise tetrameric enzyme [30], whereas the equivalent change (C105S) in rat MATα1 increased the tetramer content [32]. Moreover, C57S and C61S induced shifts towards increased rat MATα1 tetramer content, with C69S leading to dimer accumulation [32]. These behaviors could be explained by three facts: (i) the central position occupied by C69 in the β-sheet of this domain; (ii) the role exerted by the C35–C61 disulfide bond to stabilize the β-sheet in a conformation precluding dimer/tetramer exchange; and (iii) a putative change of orientation in the β-sheet due to C105 mutation at the B2–B3 loop [29]. In our case, displacement of the equilibrium towards dimer or tetramer accumulation was induced by mutations on residues located at B1–B2 (T63) or B3–C1 (E112) loops connecting β-strands of the central domain β-sheet, at β-strand B3 (N106) or at the helix α2 (Q81 and R85) placed closer to the protein surface (Figure 5) [15]. Thus, it could be postulated that dimer-prone mutations disrupt key tetramerization interactions involving T63 on the B1–B2 loop and R85 on helix α2; these interactions putatively maintain an appropriate orientation of these structural elements for dimer–dimer interaction. Such a hypothesis was further supported by results of the R85L–T63V double mutant that enhanced the effects induced by both single mutations on MAT III accumulation. On the other hand, interactions established by N106 and E112, located at the start of β-strand B3 and on the B3–C1 loop, respectively, and, in turn, at the opposite side of the β-sheet, seem to have a role precluding the tetramer-prone orientation of this structural element, because their elimination induces tetramer accumulation. Again, a residue on helix α2 (Q81), in this case located towards the B3–C1 loop, seems to contribute to the dimer-prone orientation. The importance of helix α2 in the association state is further highlighted by the effects of mutants at the R85 position, where neutral or basic residues maintain a similar MAT III/I ratio, whereas a negative charged amino acid favored dimerization. Thus, both the β-sheet (mostly probably the β-strand B2) and the helix α2 of the central domain are key for association, with slight alterations in their orientations putatively changing the oligomerization behavior of the resulting proteins.

### 2.3. Purification and Structural Characterization of the Mutant Proteins

**Figure 6:**
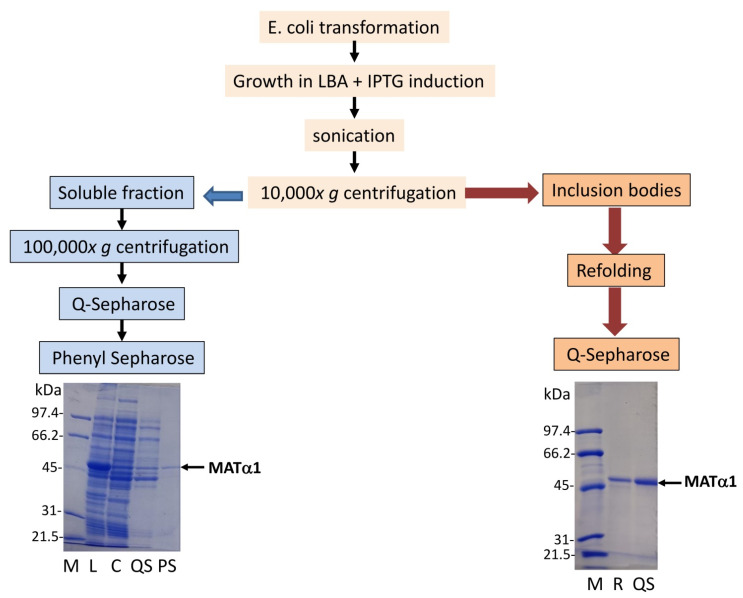
Schematic representation of the purification procedures. The figure shows the workflow used for purification of wild-type MATα1 (SSRL) and mutant proteins from soluble fractions and inclusion bodies. Representative examples of Coomassie-Blue-stained SDS-PAGE gels of each type of purification are included below. The lanes contain the markers (M), followed by samples of: lysate (L), cytosol (C), DTT-refolded protein (R), Q-Sepharose (QS) and phenyl Sepharose elution peaks (PS). The molecular weight of the markers and the MATα1 band are indicated on the left and right sides of each gel, respectively.

Purification of the mutant proteins with disrupted polar interactions at the dimer–dimer interface was required for further analysis. Successful purification of single mutants from soluble fractions was achieved in a reasonable yield after ultracentrifugation, using anion exchange and hydrophobic chromatographies (Figure 6). However, low yields allowing limited characterization (R85L–E112L) or no protein at all were obtained for double mutants that mainly accumulated in inclusion bodies. Therefore, further analysis and reliable comparisons required purification of the double mutants, as well as SSRL and single mutants, from extensively washed inclusion bodies after DTT-refolding using anion exchange chromatography. In general, the preparations obtained from either source, soluble or insoluble fractions, were >95% homogeneous, as judged by Coomassie Blue staining of the corresponding SDS-PAGE gels (Figure 6).

**Figure 7:**
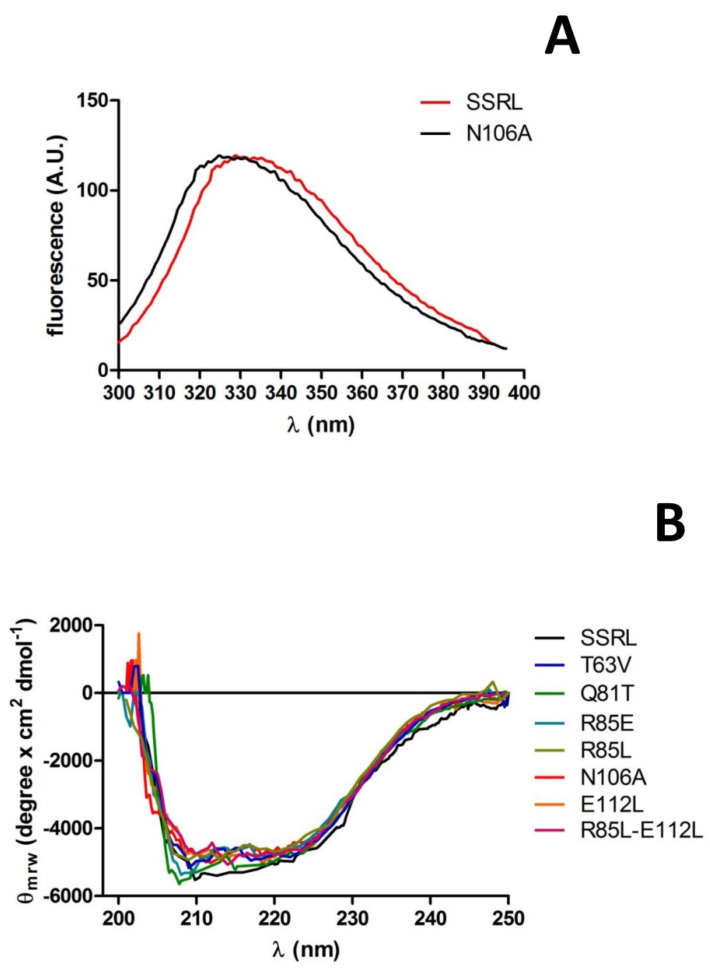
Tertiary and secondary structure analysis of wild-type MATα1 and mutants. Purified soluble proteins were used to obtain their fluorescence (50 µg/mL) and far-UV circular dichroism spectra (0.2 mg/mL). Panel (**A**) illustrates changes in fluorescence spectra (mean of 10 scans) of N106A vs. wild-type MATα1 (SSRL). Panel (**B**) shows the mean of 6 scans for each protein form after correction for background factors.

Purified soluble proteins were initially analyzed by fluorescence spectroscopy and circular dichroism to detect putative changes induced by the mutations in tertiary and secondary structure, respectively. Fluorescence spectra for most mutants and SSRL were superimposable (Figure 7A); therefore, no major changes in tertiary structure around tryptophans seem to have been produced. Only a slight blue-shift of 4 nm in the λ_max_ of N106A was detected (Figure 7A), an effect that cannot be easily explained due to its distant location to the four tryptophan residues of MATα1 according to the crystal structure. In fact, the nearest tryptophan, W176, was situated at the opposite side of the monomer and towards the surface [15]. Circular dichroism spectra of single mutants and R85L–E112L also showed no major changes when compared to SSRL (Figure 7B), although those of Q81T and R85E became slightly more negative in the 205–210 nm range, suggesting a small increase in α-helix content. Both Q81 and R85 already lay in helix α2 (D79–G92) [15], and the mutations carried out are conservative regarding α-helix propensity; the Q81T change substitutes a helix-forming with a helix-indifferent residue, and R85E does the opposite [51]. Hence, the small change in observed ellipticity could be explained by a slight length increase in this secondary structure element favored by substitutions with residues of smaller sizes. Such a slight modification was not predicted by either PSIPRED [52] or Jpred 4 [53], which calculated the same patterns of high helix propensity for SSRL, Q81T and R85E. Nevertheless, PSIPRED also calculated high helix propensity at the S96–K98 segment for the three protein forms and increased the propensity of the coil for the G99 residue in the mutant proteins. Another possibility to explain this slight change in the spectra relies on the contribution of new putative polar bonds involving E85, or the suppression of those established through Q81 amino groups, to increase or stabilize the α-helix length.

### 2.4. Effects of the Mutations on Protein Thermal Stability

**Figure 8:**
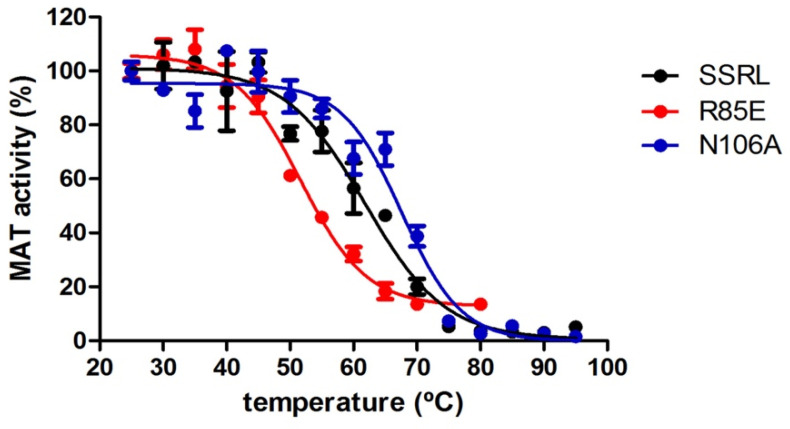
Stability of methionine adenosyltransferase proteins against temperature. The figure shows representative thermal denaturation curves of wild-type (SSRL), R85E and N106A mutant proteins purified from the soluble fraction. Samples of each protein (500 μL) at 0.1 mg/mL were incubated for 5 min at the indicated temperatures, and the remaining AdoMet synthetase activity was measured in triplicate. The values shown are the mean ± SEM of a typical experiment measured in triplicate.

Putative alterations in protein stability were evaluated by measuring the remaining AdoMet activity of purified soluble proteins incubated in the 25–90 °C range. Measurements of activity were preferred over other methods because they depend on protein oligomerization. These assays detected significant changes in the melting temperature (T_m_) of all single mutants compared to SSRL, apart from E112L (Table 3 and Figure 8). Precisely, reductions of 5–10 °C in this parameter were found for T63V, Q81T, R85L and R85E, indicating their lower thermal stability. Conversely, increases of 5 °C in the T_m_ of N106A protein were measured, revealing enhanced stability. Surprisingly, the calculated T_m_ for soluble SSRL was 10 °C above that previously reported for the DTT-refolded protein using activity measurements, fluorescence and infrared spectroscopies [54]. Comparison of their denaturation curves showed that the differences lay in the range where activity decay for refolded (37–55 °C) [54] and soluble SSRL (37–75 °C) took place. This temperature range is also where protein dissociation and unfolding of the refolded SSRL occurs [54]; hence, this increased range suggests an enhanced resistance of the soluble form towards these events. According to 2D-infrared spectroscopy, initial changes induced by temperature occur in α-helixes slightly preceding activity loss [54], a range where no differential behavior is observed between refolded and soluble SSRL proteins. Therefore, differences in protein folding/structure that may arise during both purification procedures could involve subtle alterations in other secondary structure elements that are not evident in the crystal structures reported to date or in circular dichroism spectra [55]. Additionally, the T_m_ changes induced by the mutations also suggest that minor changes in the B1–B2 loop (T63) and helix α2 (Q81 and R85) are enough to destabilize the protein. In fact, K62 and T63 belong to one of the core areas of greatest conservation within the MAT family; precisely, to block II, including sequences for β-strands B1 and B2 [10]. In contrast, Q81, R85, L111 and E112 appear in the less conserved area between blocks II and III, but L111 and E112 immediately precede the latter [10]. Moreover, those substitutions taking place on smaller and more peripheral elements contributing to the β-sheet, such as β-strand B3 (N106), seem to be better tolerated and even confer higher resistance against denaturation.

### 2.5. Kinetic Characterization of Purified Soluble MATα1 Mutants

The two MAT activities were initially analyzed using purified soluble protein forms at a concentration of 0.1 mg/mL, at which the dimer is the predominant oligomeric form [49]. Soluble SSRL showed the previously described sigmoidal dependence of AdoMet synthesis activity on methionine, ATP and Mg^2+^ concentrations, suggestive of cooperativity, whereas hyperbolic curves were found for its dependence on K^+^ concentrations [27,47,55]. These same patterns were preserved by all single mutants. The V_max_ and affinity values obtained for soluble SSRL were in the same range as that previously reported for purified rat liver MAT III, thus confirming that at the protein concentration used in the assays, the main protein form is the dimer. The mutations had different impacts in the V_max_ for AdoMet synthesis compared to SSRL, which ranged from preservation (Q81T) to decreases of 60–87% in the values for most single mutants (Table 4). As already mentioned, disruption of other polar interaction in the molecule by R265H substitution eliminated AdoMet synthesis activity, an effect that was ascribed to the monomeric association state of this mutant protein and the role of R265 in K^+^ activation [5].

On the other hand, T63V, R85L and R85E mutants showed decreased affinities for both substrates and cations, with ATP and K^+^ affinity reductions ranging approximately between 2- and 25-fold, respectively. In contrast, methionine and ATP affinities increased with the N106A and E112L mutations, whereas decreasing Mg^2+^ affinity and inducing subtle changes was observed for K^+^. A mixed pattern was exhibited by the Q81T mutant with unmodified methionine affinity, together with increased ATP, Mg^2+^ and K^+^ affinities. As mentioned before, the low purification yield obtained for soluble double mutants precluded kinetic characterization of their AdoMet synthesis activity. No obvious explanation for the described kinetic changes can be inferred from the position occupied by the mutated residues relative to the active site. None of them is directly involved in catalysis or cation binding according to the available structures [15,56]; hence, the effects must derive from indirect alterations induced by changes in the position of structural elements contributing to the active site or the loop of access. Nevertheless, most changes in methionine affinities were correlated with the association state attained by each mutant protein; methionine affinities increased in tetramer-prone mutants (N106A and E112L) and decreased in dimer-prone mutants (T63V and R85E).

Kinetic characterization of the PPPase activity of soluble purified proteins was also carried out at the same protein concentration in the presence or absence of AdoMet. According to previous reports, our soluble SSRL showed 18- and 4-fold higher PPPase activity than its human His-tagged counterpart [39] and purified rat liver MAT III [57], respectively. Such differences could arise from the distinct purification protocols or the presence of a tag which, depending on the position and type, could affect the folding, association and/or activity of MATs [12,18,58]. Dependence on PPP_i_ concentrations in the presence or absence of AdoMet were characterized by hyperbolic curves for SSRL and all the mutants, in contrast to the slightly sigmoidal curves previously described for purified rat liver MAT III [57]. Moreover, SSRL showed 2.5- and 5-fold increases in V_max_ and PPP_i_ affinity in the presence of AdoMet, respectively (Table 5). Stimulation of PPPase activity by the methyl donor was reported in studies of MATs from different origins [3,59,60,61], the induction taking place in the 50–100 µM range for rat liver MATs [59]. Nevertheless, no changes in the PPPase kinetic parameters of purified rat liver MAT III were more recently reported [57], differences that may depend on the AdoMet concentrations used in each study (5 mM vs. 50 µM in our work). It is well known that AdoMet synthesis activity is inhibited by AdoMet itself, the effects on MAT I and MAT III being exerted at quite different concentrations [47]; however, whether a similar behavior occurs on PPPase activity has not been explored [59].

Regarding the PPPase activity of the mutants, in the absence of AdoMet, most of them displayed increased V_max_ values compared to SSRL which reached 2- and 3.3-fold for R85L and E112L proteins, respectively. Surprisingly, the double R85L–E112L mutant displayed enough PPPase activity for its kinetic characterization, the calculated V_max_ being slightly decreased compared to that of SSRL. Preservation of PPPase activity seems common to mutants disrupting polar interactions, independently of their association state, as suggested from its conservation in the monomeric rat R265H mutant [5]. On the other hand, the stimulatory effect of AdoMet was only preserved in the E112L and R85L–E112L mutants, whereas addition of the methyl donor decreased the V_max_ of R85L PPPase activity. Additionally, all mutants displayed increased affinity for PPP_i_ in the absence of AdoMet, this effect being more important in the double R85L–E112L mutant. The methyl donor modestly increased substrate affinity in most mutant proteins, but decreased that of E112L and R85L–E112L proteins. Again, the position of the mutated residues relative to the catalytic site cannot directly explain these effects [15,56], which once more could rely on slight modifications in the orientation of secondary structure elements contributing to catalysis and/or substrate binding.

### 2.6. Kinetic Characterization of Purified Refolded Double Mutants and Comparison with Wild-Type SSRL and Single Mutants

**Figure 9:**
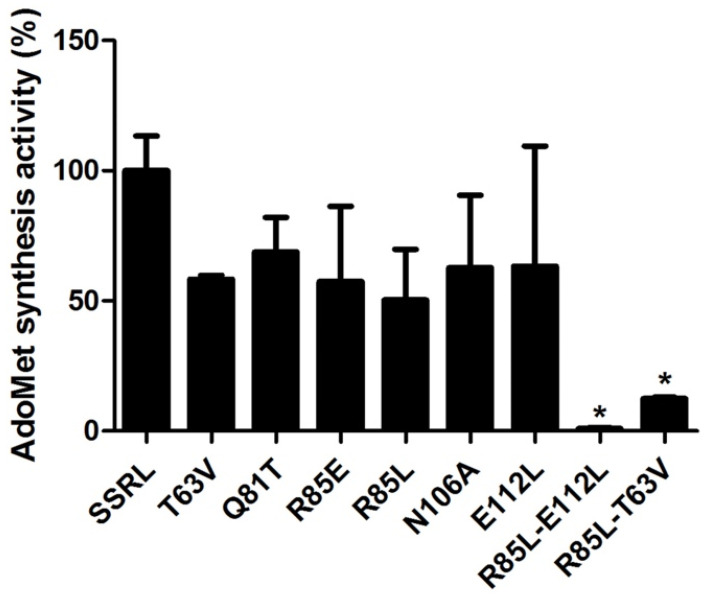
Total AdoMet synthetase activity of purified DTT-refolded methionine adenosyltransferase proteins. Pellets of inclusion bodies with the same size were used for the refolding and purification of wild-type (SSRL) and mutant methionine adenosyltransferases. Protein concentration and AdoMet synthetase activities were measured to calculate the protein yield and total activity recovered by this procedure. The figure shows the percentage of total AdoMet synthetase activity calculated vs. SSRL (mean ± SEM) of at least two independent batches for each protein measured in triplicate. In all cases, the protein yield was similar. Results were considered significant vs. SSRL when *p* ≤ 0.05 (*).

In order to attain enough protein to kinetically characterize double mutants, DTT-refolding from inclusion bodies was performed, a procedure also carried out for SSRL and single mutant proteins to allow comparison between protein forms obtained under the same conditions. Starting from pellets of inclusion bodies of similar size, the amount of purified refolded protein recovered was comparable for SSRL and all mutants, achieving 6–8.4 mg per batch. In this case, all the refolded mutant proteins exhibited decreased AdoMet synthesis activity, including Q81T, although high variability between batches was detected (Figure 9). This effect was exacerbated in the double mutants with <10% of the AdoMet synthesis activity exhibited by SSRL (Figure 9).

AdoMet synthesis activity of refolded SSRL and single mutants showed sigmoidal dependence against methionine and ATP concentrations, as described above for the purified soluble proteins. Moreover, the kinetic data obtained for refolded SSRL were very similar to those previously reported [55]. Comparison of V_max_ values of the refolded mutants with those calculated for their purified soluble forms showed comparable patterns with decreases for all single mutants, apart from Q81T (Table 6). Regarding methionine and ATP affinities, T63V, R85L and R85E exhibited important reductions, as already described for the corresponding soluble proteins, but differences were detected in the behavior of N106A and E112L mutants. The refolded N106A protein still increased its affinity for methionine, but decreased that for ATP, whereas the affinity for both substrates was reduced in the E112L refolded protein. Once more, refolded double mutants had very low AdoMet synthetase activity for allowing kinetic characterization. Again, methionine affinities of the mutants roughly correlated with the changes induced in their association state; dimer-prone mutants (T63V and R85E) decreased their affinities, whereas increased (N106A) or no change (Q81T) in affinity was detected for tetramer-prone substitutions.

In contrast to the effects on AdoMet synthesis, all the refolded mutants exhibited PPPase activity, at least similar to that of SRRL, with no significant changes in PPP_i_ affinities in the absence of AdoMet (Table 7). The kinetic curves also preserved the hyperbolic shape described for soluble proteins. AdoMet addition did not stimulate the PPPase activity of refolded SSRL, but decreased its affinity for PPP_i_ 4.5-fold (Table 7). Lack of AdoMet stimulation was also found for the refolded mutants, with addition of the methyl donor even reducing the PPPase activity of T63V, Q81T, R85E and R85L–E112L proteins. In the presence of AdoMet, most mutants exhibited higher affinities for PPP_i_ than SSRL, apart from R85L and R85L–T63V. Moreover, PPP_i_ affinities of the mutants were decreased through addition of the methyl donor, but these changes were, in general, more modest than for refolded SSRL. Exceptions to this rule were R85L and R85L–T63V proteins, for which the impact on PPP_i_ affinity was similar to that found for SSRL. Comparison of the effects on PPPase kinetics is precluded by a lack of information for other MAT mutants in this region, as previously mentioned.

### 2.7. Concluding Remarks

Disruption of the few polar interactions of <3.5 Å at the dimer–dimer interface of rat MAT I exerts diverse effects which are not only restricted to tetramerization, but also affect protein stability, AdoMet synthesis and PPPase activities. Mutations favoring dimer formation, especially those on R85 and T63 residues, also induce kinetic changes similar to those previously described between MAT I and MAT III proteins, namely, decreased substrate affinities. Double mutants erase AdoMet synthesis activity while preserving PPPase activity and exacerbating dimer accumulation, highlighting the importance of polar interactions involving T63, R85 and E112 to sustain dimer–dimer association in MAT I. These three residues locate on the central domain of MATα1 subunits on loops at opposite sides of the β-sheet or on helix α2 located behind, towards the protein surface. Moreover, E112 is placed on a coiled-coil segment preceding the loop of access to the active site, a fact that may explain effects exerted by its substitution on activity putatively due to alterations altering the entrance to the catalytic site. Kinetic effects induced by other mutants have no evident explanation and may derive from their impact on the stability of the corresponding structural elements or to changes in their conformations/orientations indirectly affecting the active site. Despite the importance of the central domain in MATα1 association state, and hence in AdoMet synthesis, the impact of human mutations on AdoMet production has scarcely been evaluated and no in-depth characterization has been carried out for any of the mutants identified to date. Although the high conservation in sequence and crystal structures exhibited by MATs, and especially among mammalian counterparts, allows inference of the impact of rat mutants on the human protein behavior, there is a need for thoughtful analyses of human substitutions that may render unexpected results.

## 3. Materials and Methods

### 3.1. Site-Directed Mutagenesis

The pSSRL-T7N plasmid reported by Álvarez et al. [36], which includes the rat MATα1 ORF, was used for the mutagenesis of residues involved in polar interactions (<3.5 Å) at the MAT I dimer–dimer interface with the QuikChange method (Stratagene, La Jolla, CA, USA) and the sense and antisense oligonucleotides shown in Table 8. Introduction of the desired modifications was verified by automatic sequencing of the corresponding plasmids at the Genomic Service of the Instituto de Investigaciones Biomédicas Alberto Sols (CSIC-UAM).

### 3.2. Heterologous Expression and Preparation of Cell Extracts

Competent *E. coli* BL21(DE3) cells were transformed with either pSSRL-T7N [36] or the corresponding mutant plasmids and grown overnight in LB plates containing 50 μg/mL ampicillin (LBA) at 37 °C. Single colonies were then used to inoculate 500 mL of LBA medium and cultures grown at 37 °C until A_595_ reached 0.3–0.4. At this point, isopropyl β-D-galactopyranoside (IPTG; AMBION, Austin, TX, USA) was added to a final concentration of 0.5 mM and incubations continued for a further 3 h at 37 °C. Bacterial pellets were obtained by centrifugation and washed with water prior to storage at −80 °C. At the time of use, the pellets were resuspended in 50 mM Tris/HCl pH 8, 10 mM MgSO_4_, 5 mM EDTA containing 0.1% (*v*/*v*) 2-mercaptoetanol (Merck, Darmstadt, Germany) and protease inhibitors (2 μg/mL aprotinin, 1 μg/mL pepstatin A, 0.5 μg/mL leupeptin, 2.5 μg/mL antipain, 0.5 mM benzamidine, 0.5 mM PMSF; Sigma, St. Louis, MO, USA) and disrupted by sonication on ice (eight 30 s pulses at 30 s intervals, output power level 8) using a Soniprep 150 sonifier. Soluble and insoluble fractions (inclusion bodies) were separated by centrifugation at 10,000× *g* for 15 min at 4 °C and the cytosol obtained by ultracentrifugation of the soluble fraction at 100,000× *g* for 45 min at 4 °C.

### 3.3. AdoMet Synthesis Measurements and Kinetics

Aliquots (160 µL) of the soluble fractions, cytosols and fractions obtained from purification steps and phenyl Sepharose dimer/tetramer analysis were used to measure AdoMet synthesis activity for 30 min at 37 °C, as described by Gil et al. [62], using 90 µL of a stock reaction mixture containing 75 mM Tris/HCl pH 8, 250 mM KCl, 9 mM MgCl_2_, 10 mM DTT, 5 mM methionine and 5 mM [2–^3^H]-ATP (4 Ci/mol). Kinetic characterization of purified proteins (0.1 mg/mL) was performed by changing substrate or cation concentrations in the stock reaction mixture using the following ranges: (i) 1 μM to 10 mM methionine at 10 mM ATP; (ii) 10 μM to 10 mM ATP at 10 mM methionine; (iii) 500 μM to 360 mM KCl at 10 mM of either substrate; (iv) 50 μM to 36 mM MgCl_2_ at 10 mM of either substrate.

### 3.4. Tripolyphosphatase Activity and Kinetics

Aliquots (100 µL) of the purified proteins (0.1 mg/mL) were also used to measure the PPPase activity of SSRL and mutant MATs in a final volume of 500 µL, in the presence or absence or 50 μM AdoMet, as previously described [15]. The reaction was initiated by the addition of 100 µL of a stock reaction mixture containing 50 mM Tris/HCl pH 7.8, 100 mM KCl, 7 mM MgCl_2_, 1 mM DTT and 2 mM PPP_i_ and incubated for 10 min at 30 °C. PPP_i_ concentrations in the reaction mixture between 50 μM and 5 mM were used in kinetic assays.

### 3.5. Evaluation of the Dimer/Tetramer Ratios by Analytical Gel Filtration and Phenyl Sepharose Chromatographies

Soluble fractions were used to determine dimer/tetramer ratios using standard protein concentrations of 0.5 mg/mL. Separation of both oligomeric forms by analytical gel filtration chromatography (AGFC) was carried out by injection of the soluble fractions (100 μL) on a Superose 12 10/30 HR column (GE Healthcare, Barcelona, Spain) equilibrated and run with 50 mM Tris/HCl pH 8, 10 mM MgSO_4_, 1 mM EDTA (buffer A) containing 150 mM KCl at 0.3 mL/min. Fractions (300 μL) were collected and used to detect MAT oligomers by dot-blot (50 µL/well). In certain cases, dimer and tetramer peaks were collected and samples of each (50 µL/lane) were loaded on SDS-PAGE gels for immunoblotting. The standards (GE Healthcare and Sigma) used for column calibration were blue dextran (2000 kDa), apoferritin (443 kDa), β-amylase (200 kDa), alcohol dehydrogenase (150 kDa), carbonic anhydrase (29 kDa), lysozyme (14.3 kDa) and ATP (551 Da). The K_AV_ value for each standard was obtained using the formula K_AV_ = (V_e_ − V_ex_)/(V_f_ − V_ex_), where V_e_ is the elution volume of the protein, and V_f_ and V_ex_ are the final and exclusion volumes of the column, respectively. M_r_ estimations were obtained using the calibration curve derived by representing the log M_r_ of the standards against their K_AV_.

In parallel, 2 mL of the same soluble fractions were loaded on phenyl Sepharose (GE Healthcare) columns (3 mL) equilibrated in buffer A, washed with 20 mL of this same buffer, and eluted with 10 mL of buffer A containing 50% (*v*/*v*) DMSO [32]. The flowthrough (2 mL) and fractions (5 mL) were collected and assayed for detection of MAT activity and MATα1 protein by dot-blot; MAT I tetramers do not bind to phenyl Sepharose and were recovered in the flowthrough, whereas MAT III dimers were eluted with DMSO [48].

### 3.6. Dot-Blot Localization of the Proteins

Dot-blotting was performed as previously described [29]. Briefly, aliquots of AGFC (50 μL) and phenyl Sepharose (100 μL) fractions were spotted on nitrocellulose membranes and denatured with 6 M guanidinium chloride (Merck). Membranes were blocked with low-fat dry milk (3% *w*/*v*) and incubated sequentially with anti-MATα1 antisera (1:10,000 *v*/*v*) prepared in our laboratory [32] and a secondary anti-rabbit-HRP antibody (1:10,000 *v*/*v*; BioRad, Hercules, CA, USA). The protein signal was then visualized with Western Lightning^TM^ chemiluminescence reagent (Perkin Elmer, Waltham, MA, USA). Quantification of dot-blots was performed by densitometric scanning of the images using ImageJ v1.37 software, and values were corrected for differences in fraction volumes.

### 3.7. Electrophoresis and Immunoblotting

Protein samples (15–35 µg) of the different purification steps were loaded on 10% SDS-PAGE gels and stained with Coomassie Blue. For immunoblotting, dimer and tetramer peaks (50 µL/lane) obtained by AGFC were loaded in the gel for electrotransference to nitrocellulose membranes, which were incubated with anti-MATα1 antiserum and visualized as described above for dot-blots. Quantification of stained gels and immunoblotting bands was performed by densitometric scanning of the images using ImageJ software.

### 3.8. Purification of Soluble Wild-Type and Mutant Proteins

Cytosols obtained from 500 mL cultures were used for purification using HiTrap columns (GE Healthcare). Samples were first injected on Q-Sepharose cartridges (5 mL) equilibrated in buffer A at 1 mL/min, washed with 5 volumes of this buffer (until A_280_ = 0), and eluted with a gradient (10 column volumes) from 0 to 1.5 M KCl in buffer A. Fractions (2 mL) were collected for activity measurements and those exhibiting AdoMet synthesis were pooled. Active peaks were then loaded on phenyl Sepharose cartridges (5 mL) equilibrated in buffer A containing 300 mM KCl at 1 mL/min, washed with 5 volumes of this buffer, and eluted with 2 bed volumes of buffer A containing 50% (*v*/*v*) DMSO. Fractions (4 mL) were collected and assayed for AdoMet synthesis activity. The active fractions were pooled and extensively dialyzed against buffer A for DMSO elimination. Dialyzed proteins were concentrated through YM-30 membranes (AMICON Corp., Beverly, MA, USA) before use. Samples of each purification step were loaded on 10% SDS-PAGE gels and stained with Coomassie Blue to assess purity.

### 3.9. Refolding and Purification of Wild-Type and Mutant Proteins from Inclusion Bodies

Inclusion bodies were washed and used for DTT-refolding of the proteins following the previously described protocol [55]. Refolded proteins were then purified on Q-Sepharose cartridges (5 mL), as described for the soluble proteins and the active fractions, pooled, and dialyzed against buffer A to eliminate excess KCl. The quality of the procedure was assessed by Coomassie Blue staining of samples loaded on 10% SDS-PAGE gels.

### 3.10. Circular Dichroism

Analysis of the secondary structure was carried out by far-UV circular dichroism in a Jasco J-700 spectropolarimeter using purified proteins (0.2 mg/mL) and 0.1 cm pathlength cuvettes. The spectra shown are the mean of 6 scans for each sample after correction for background factors and conversion of the observed ellipticities into mean residue ellipticities (Ɵ_mrw_) using a mean molecular mass per residue of 110 Da.

### 3.11. Fluorescence Emission Spectra

The impact of mutations on the tertiary structure around tryptophans was evaluated by measuring the intrinsic fluorescence of the DTT-refolded purified proteins (50 µg/mL) upon excitation at 295 nm (slit width 2.5 nm). A minimum of 10 emission spectra were recorded between 300 and 400 nm (slit width 5 nm) in a photon-counting SLM-8000 spectrofluorometer at 25 °C using 0.5 × 0.5 cm cuvettes and corrected for background factors.

### 3.12. Protein Stability

Samples (500 µL) of purified SSRL and mutant MATs at 0.1 mg/mL were incubated for 5 min at temperatures in the 25–80 °C range, although in some cases this range had to be extended to 95 °C. The remaining AdoMet synthesis activity was measured in triplicate as described above, and the data were used to calculate the melting temperatures (T_m_) using GraphPad Prism v5.0 software.

### 3.13. Measurements of Distances between Residues and Secondary Structure Prediction

The previously reported rat MAT I crystal structure (1QM4) [15] and Swiss-PdbViewer v4.1.0 software were used to measure distances between residues of interest, whereas figures were prepared with PyMol v2.0 software (Schrödinger, LLC, New York, NY, USA). Secondary structure predictions were performed on the PSIPRED [52] and Jpred 4 [53] servers using the wild-type or mutated rat MATα1 sequences.

### 3.14. Protein Concentration Determinations

Samples at each purification and fractionation steps were taken to measure protein concentrations using the BioRad protein assay kit [63]. Purified protein concentrations were determined spectrophotometrically using a calculated molar extinction coefficient at 280 nm of 41,934 M^−1^cm^−1^ in 8 M urea.

### 3.15. Statistical Analysis

Student’s *t*-tests for unpaired samples were performed using GraphPad Prism v5.0 (GraphPad Software, San Diego, CA, USA) and differences were considered significant when *p* ≤ 0.05.

## Figures and Tables

**Table 1:** Dimer–dimer polar interactions established at <3.5 Å.

Monomer A	Atom	Monomer C	Atom	Monomer D	Atom	Distance (Å) ^1^
Q81 ^2^	NE	L111	O	-	-	3.45
Q81 ^2^	OE1	E112 ^2^	OE2	-	-	2.81
R85	NH2	E112 ^2^	OE2	-	-	2.76
K62	O	-	-	N106 ^2^	OD1	3.15
T63	O	-	-	N106 ^2^	OD1	3.27

^1^ Distances measured using Swiss-PdbViewer v4.10.; ^2^ Residues involved in two polar interactions each.

**Table 2:** AdoMet synthesis activity in the soluble fraction.

	AdoMet Synthesis Activity (nmol/min/mg) ^1^		Activity Induction Fold
	−IPTG	+IPTG	
SSRL	0.25 ± 0.03	15.16 ± 0.25	60.64
T63V	0.15 ± 0.01	0.93 ± 0.14	6.2
Q81T	0.35 ± 0.01	17.91 ± 0.48	51.17
R85E	0.19 ± 0.02	2.1 ± 0.21	11.05
R85L	0.25 ± 0.01	3.16 ± 0.24	12.64
N106A	0.4 ± 0.01	4.17 ± 0.12	10.42
E112L	0.22 ± 0.01	1.21 ± 0.12	5.5
R85L–E112L	0.23 ± 0.02	0.72 ± 0.19	3.13
R85L–T63V	0.28 ± 0.03	0.35 ± 0.24 ^2^	-

^1^ The values shown are the mean ± SD of three experiments carried out in triplicate; ^2^ not significant.

**Table 3:** Protein stability assayed by the thermal denaturation of purified soluble methionine adenosyltransferase forms.

	T_m_ (°C) ^1^	P vs. SSRL
SSRL	61.83 ± 1.11	-
T63V	52.39 ± 3.13	0.0082
Q81T	53.55 ± 2.09	0.0037
R85E	51.73 ± 1.12	0.0004
R85L	57.01 ± 2.01	0.022
N106A	67.64 ± 0.84	0.0019
E112L	64.18 ± 1.25	0.074 ^2^

^1^ The values shown are the mean ± SD of two independent experiments carried out in triplicate; ^2^ non-significant vs. SSRL.

**Table 4:** Kinetic data of the AdoMet synthesis activity from purified soluble *wild-type* methionine adenosyltransferase (SSRL) and mutants ^1^.

	S_0.5_			K_m_	V_max_
	Met (μM)	ATP (μM)	Mg^2+^ (mM)	K^+^ (mM)	(nmol/min/mg)
SSRL	309.3 ± 3.17	791.7 ± 52.4	4.05 ± 0.34	2.55 ± 0.39	45.36 ± 7.34
T63V	2460 ± 180	1033 ± 70	7.46 ± 0.44	56.87 ± 13.23	6.21 ± 1.91
Q81T	301.5 ± 28.8	478.8 ± 24.9	2.5 ± 0.14	1.89 ± 0.15	55.5 ± 0.45
R85E	2420 ± 520	1420 ± 120	5.39 ± 0.29	41.4 ± 10.01	10.79 ± 3.21
R85L	2120 ± 160	1920 ± 100	7.37 ± 0.35	50.43 ± 9.33	18.96 ± 6.91
N106A	40.64 ± 8.64	266.4 ± 31.17	6.24 ± 0.64	1.52 ± 0.22	14.13 ± 2.89
E112L	114.1 ± 30.2	215.6 ± 27.4	8.34 ± 0.66	3.3 ± 0.97	8.17 ± 0.37

^1^ The values shown are the mean ± SEM of a minimum of two experiments carried out in triplicate.

**Table 5:** Kinetics of the tripolyphosphatase activity of soluble wild-type methionine adenosyltransferase (SSRL) and mutants ^1^.

	−AdoMet		+AdoMet	
	V_max_ (μmol/min/mg)	K_m_^PPPi^ (mM)	V_max_ (μmol/min/mg)	K_m_^PPPi^ (mM)
SSRL	0.63 ± 0.04	2.52 ± 0.3	1.65 ± 0.07	0.48 ± 0.06
T63V	1.05 ± 0.04	1.52 ± 0.1	0.91 ± 0.03	0.89 ± 0.07
Q81T	0.86 ± 0.04	2.28 ± 0.19	0.76 ± 0.08	1.44 ± 0.3
R85E	0.91 ± 0.06	1.77 ± 0.22	0.83 ± 0.09	1.25 ± 0.25
R85L	1.24 ± 0.04	2.13 ± 0.12	0.92 ± 0.04	1.22 ± 0.11
N106A	0.81 ± 0.03	1.58 ± 0.11	0.89 ± 0.04	1.17 ± 0.11
E112L	2.09 ± 0.06	1.59 ± 0.08	3.31 ± 0.12	2.4 ± 0.15
R85L–E112L	0.47 ± 0.04	0.78 ± 0.14	1.11 ± 0.03	1.12 ± 0.07
R85L–T63V	-	-	-	-

^1^ The values shown are the mean ± SEM of at least two experiments carried out in triplicate.

**Table 6:** Kinetics of AdoMet synthesis activity of DTT-refolded proteins ^1^.

	V_max_ (nmol/min/mg)	S_0.5_^Met^ (μM)	S_0.5_^ATP^ (μM)
SSRL	109.7 ± 29.9	295.13 ± 19.9	407.35 ± 32.3
T63V	54.95 ± 3.24	1670 ± 70	2050 ± 30
Q81T	112.9 ± 0.41	237.34 ± 0.14	481.3 ± 7.9
R85E	51.58 ± 2.26	1270 ± 120	1760 ± 98
R85L	59.45 ± 4.0	1580 ± 107	1690 ± 45
N106A	46.76 ± 0.02	29.5 ± 0.02	735.8 ± 126.5
E112L	55.05 ± 2. 59	430 ± 38.3	1170 ± 39
R85L–E112L	nd ^2^	nd	nd
R85L–T63V	nd	nd	nd

^1^ The values shown are the mean ± SEM of a typical experiment carried out in triplicate; ^2^ nd, non-detectable.

**Table 7:** Kinetics of tripolyphosphatase activity of DTT-refolded proteins ^1^.

	−AdoMet		+AdoMet	
	V_max_ (μmol/min/mg)	K_m_^PPPi^ (mM)	V_max_ (μmol/min/mg)	K_m_^PPPi^ (mM)
SSRL	0.86 ± 0.09	0.22 ±0.1	0.83 ± 0.05	0.97 ± 0.12
T63V	1.31 ± 0.08	0.29 ± 0.06	1.01 ± 0.05	0.48 ± 0.07
Q81T	1.54 ± 0.06	0.25 ± 0.03	1.05 ± 0.04	0.32 ± 0.04
R85E	1.24 ± 0.06	0.26 ± 0.04	0.75 ± 0.04	0.49 ± 0.08
R85L	1.19 ± 0.07	0.27 ± 0.05	0.98 ± 0.05	0.97 ± 0.1
N106A	1.04 ± 0.05	0.27 ± 0.04	0.97 ± 0.04	0.55 ± 0.06
E112L	0.94 ± 0.04	0.16 ± 0.03	1.08 ± 0.03	0.24 ± 0.03
R85L–E112L	1.22 ± 0.08	0.37 ± 0.07	0.79 ± 0.03	0.56 ± 0.06
R85L–T63V	0.96 ± 0.06	0.31 ± 0.06	1.08 ± 0.08	0.87 ± 0.14

^1^ The values shown are the mean ± SEM of a typical experiment carried out in triplicate.

**Table 8:** Mutagenic oligonucleotides.

Mutation	Oligonucleotide Sequence ^1^	
T63V	5′-GACAGTGTGCAAG**GT**AGGGATGGTGCTC-3′ 5′-GAGCACCATCCCTACCTTGCACACTGTC-3′	Sense antisense
Q81T	5′-CCATGATTGACTAC**AC**GCGGGTGGTGAGAGAC-3′ 5′-GTCTCTCACCACCCGCGTGTAGTCAATCATGG-3′	Sense antisense
R85E	5′-CCAGCGGGTGGTG**GA**AGACACCATTAAGC-3′ 5′-GCTTAATGGTGTCTTCCACCACCCGCTGG-3′	Sense antisense
R85L	5′-CCAGCGGGTGGTG**CT**AGACACCATTAAGCA-3′ 5′-TGCTTAATGGTGTCTAGCACCACCCGCTGG-3′	Sense antisense
N106A	5′-ACTTCAAGACCTGC**GC**TGTGCTCGTGGCTC-3′ 5′-GAGCCACGAGCACAGCGCAGGTCTTGAAGT-3′	Sense antisense
E112L	5′-GCTCGTGGCTCTG**CT**GCAACAGTCCCCAG-3′ 5′-ACTTCAAGACCTGCGCTGTGCTCGTGGCTC-3′	Sense antisense

^1^ Nucleotide changes appear in bold and underlined in the sense oligonucleotide sequence.

## Data Availability

Data are available upon request from the corresponding author. Original images of the stained gels shown in Figure 2A,B and Figure 6, as well as raw CD data, are provided as Supporting Information.
